# Pharmacokinetic profile and *in vivo* anticancer efficacy of anagrelide administered subcutaneously in rodents

**DOI:** 10.1080/10717544.2025.2463433

**Published:** 2025-02-10

**Authors:** Kirsi Toivanen, Luna De Sutter, Agnieszka Wozniak, Karo Wyns, Nanna Merikoski, Sami Salmikangas, Jianmin Duan, Mikael Maksimow, Maria Lahtinen, Tom Böhling, Patrick Schöffski, Harri Sihto

**Affiliations:** a Department of Pathology, University of Helsinki and Helsinki University Hospital, Helsinki, Finland; b Department of Oncology, Laboratory of Experimental Oncology, KU Leuven, Leuven Cancer Institute, Leuven, Belgium; c Duan Pharmaceutical Consulting Inc., Laval, Canada; d Sartar Therapeutics Ltd., Helsinki, Finland

**Keywords:** Anagrelide, subcutaneous, slow-release, pharmacokinetics, GIST

## Abstract

Anagrelide (ANA) is a phosphodiesterase 3A (PDE3A) inhibitor, commonly prescribed for essential thrombocythemia. It also functions as a molecular glue, inducing complex formation between PDE3A and Schlafen 12. This association either triggers apoptosis or inhibits proliferation in tumor cells, supporting its use in cancer therapy. Conventionally administered orally, ANA undergoes rapid metabolism and elimination, resulting in a short drug exposure time at the site of action. Here, we explored the pharmacokinetic profile of a subcutaneously (SC) injected ANA formulation in Sprague-Dawley rats by quantifying plasma ANA and metabolite concentrations using liquid-chromatography–tandem mass spectrometry. We further evaluated the *in vivo* tumor regression efficacy of orally and SC administered ANA in a patient-derived gastrointestinal stromal xenograft mouse model – UZLX-GIST2B – characterized by a *KIT* exon 9 driver mutation. The SC ANA exhibited extended-release plasma concentration–time profiles compared to intravenous and oral administrations. After a single administration in rats, plasma concentrations of ANA were detected up to 56 days later, and ANA metabolites up to 30 days later. The SC formulation also significantly reduced tumor volumes and demonstrated dose-dependent histological responses, nearly eradicating tumor tissue in 11 days with the highest dose. These findings suggest that the SC slow-release formulation maintains stable drug concentrations during treatment, potentially improving therapeutic efficacy at the target site.

## Introduction

Anagrelide (ANA, imidazo‐[2,1‐b] quinazolin‐2[3H]‐one, 6,7‐dichloro‐1,5‐dihydro, monohydro‐chloride) is an inhibitor of the phosphodiesterase (PDE) 3 enzyme. PDEs degrade cyclic nucleotides, which act as second messengers in numerous cellular signaling pathways (Conti & Beavo, 2007). In addition to ANA’s inhibitory role, it also exhibits a unique capability as a ‘molecular glue’ between phosphodiesterase 3A (PDE3A) and Schlafen 12 (SLFN12), which leads to toxicity in cells that express both proteins (An et al., 2019; Toivanen et al., 2023). Compounds with this ability are referred to as PDE3A modulators or velcrins (de Waal et al., 2016; Lewis et al., 2019; Li et al., 2019; Garvie et al., 2021; Yan et al., 2022; Lee et al., 2023). These compounds are considered for oncological applications due to their observed antitumor effects *in vitro* across various cell lines, including gastrointestinal stromal tumor (GIST), liposarcoma, neuroglioma, hepatocellular carcinoma, melanoma, ovarian carcinoma, and cervical adenocarcinoma (Nazir et al., 2017; An et al., 2019; Pulkka et al., 2019; Toivanen et al., 2023). *In vivo* studies in mice inoculated with cervical cancer HeLa (Chen et al., 2021) and neuroglioma H4 (An et al., 2019) cells, and with patient-derived GIST tumors (Pulkka et al., 2019) further support the therapeutic potential of ANA. ANA is an approved drug for treating essential thrombocythemia, as it inhibits megakaryocyte development and reduces platelet number (Spencer & Brogden, 1994; Petrides et al., 2018), which is suggested to be independent of PDE3 inhibition (Wang et al., 2005; Espasandin et al., 2015; Chen et al., 2021).

Traditionally taken orally (per os = PO), ANA is absorbed via the gastrointestinal tract with a bioavailability of approximately 70%; in the liver, it is metabolized to two main metabolites − 3′-hydroxy-anagrelide (3-OH-ANA) and 2‐amino‐5,6‐dichloro‐3,4‐dihydroquinazolin (RL603) (Spencer & Brogden, 1994; Petrides et al., 2009, 2018). 3-OH-ANA is considered an active metabolite, exhibiting potent inhibition of megakaryocyte development. On the other hand, evidence suggests that RL603 is a less active metabolite (Lane et al., 2001; Erusalimsky et al., 2002; Wang et al., 2005), although findings have been controversial. However, the potential of these metabolites to act as molecular glues between PDE3A and SLFN12 remains unexplored, despite the fact that ANA drug sensitivity in tumor cells is thought to mostly correlate with these protein expression levels (Nazir et al., 2017; An et al., 2019).

Peak plasma concentration of ANA and its metabolites is observed approximately one hour after PO drug administration, with plasma half-lives (*T*_1/2_) ranging from 1.5 to 2.5 h (Gaver et al., 1981; Petrides et al., 2009, 2018). Although ANA absorption and bioavailability can be improved under fed conditions, the metabolites are eliminated relatively fast from the body, becoming nearly undetectable within 24 h from the dosage (Martínez-Sellés et al., 2013; Petrides et al., 2018). Consequently, efficient treatment of essential thrombocythemia requires frequent administration, causing repeated high plasma peaks that may result in more adverse events (e.g. dizziness, headache, and heart palpitations) (Petrides et al., 2009, 2018).

To improve the pharmacokinetic (PK) features of water-insoluble and very fast turnover compounds such as ANA, extended-release formulations have been developed. For ANA, a polyacrylic acid-based tablet with extended-release ability has been found to be as effective in lowering platelet counts in patients with essential thrombocythemia as compared to the commercially available formulation (Petrides et al., 2018; Gisslinger et al., 2019). Notably, it also decreases the occurrence of adverse events, particularly palpitations (Petrides et al., 2018). No other sustained-release delivery options for ANA have been reported.

Examples of other extended-release formulations include nanostructured lipid carriers (Zhang et al., 2013), hydrogels (Phan et al., 2016), multivesicular liposomes (Qiu et al., 2005), hydrogel microparticles (Hadidi & Pazuki, 2022), and microspheres (Zhang et al., 2008; Wang et al., 2021), which have demonstrated promising results in drug delivery. Lipid-based delivery systems such as miglyol oil vehicles, are mixtures of medium-chain triglycerides that are considered safe, as they have been widely studied in various organisms with no reported adverse events (Palin et al., 1982; Wicks et al., 1993; Wysham et al., 2016; Buss et al., 2018; Aparna et al., 2021; Corciulo et al., 2021; Subhramanian et al., 2023). Using miglyol or miglyol-based carriers has been shown to lead to significantly improved therapeutic effects, also for compounds with short systemic exposures or poor water solubility, or both (Wicks et al., 1993; Zhang et al., 2013; Corciulo et al., 2021; Subhramanian et al., 2023). The administration route also contributes to increased drug absorption, bioavailability, and metabolism. PO administration is often considered the most convenient option, as it requires less intensive monitoring by healthcare professionals once dosing and side effects are evaluated and managed effectively, unlike intravenous (IV) administration, However, subcutaneous (SC) drug delivery may offer a compromise between PO and IV administration. With proper training and instruction from healthcare professionals, SC injections can be done by patients themselves. Furthermore, when delivered via the SC route, compounds bypass liver metabolism, reducing elimination rate and resulting in improved bioavailability, potentially altering systemic distribution patterns and prolonging the effective *T*_1/2_, thereby reducing the need for frequent dosing (Alexander-Curtis et al., 2017; Klose et al., 2022).

Here, we chose SC as the administration route and miglyol (810 N) as a vehicle for ANA, given its effectiveness in dissolving poorly water-soluble compounds and its recognized safety as a commercially available lipid vehicle (Palin et al., 1982; Wicks et al., 1993; Zhang et al., 2013; Wysham et al., 2016; Buss et al., 2018). We compared PK profiles across three administration routes (SC, PO, and IV) and investigated the long-term sustained-release in rats. Finally, we evaluated the therapeutic impact of SC ANA in a patient-derived GIST xenograft mouse model, UZLX-GIST2B, that has previously demonstrated sensitivity to ANA (Pulkka et al., 2019) and two tyrosine kinase inhibitors, imatinib and sunitinib (De Sutter et al., 2023). The objective was to establish an administration option with extended-release PK characteristics and evaluate its anti-tumor efficacy.

## Materials and methods

### Study design

To compare PK values of the three different administration routes, 11 male Sprague-Dawley^®^ (SD) rats (6–8 weeks old) were administered a single dose of ANA either via IV injection (*n* = 3, 0.5 mg/kg), PO (*n* = 3, 2.5 mg/kg), or SC injection (*n* = 5, 1.0 mg/kg). To monitor the sustained release of SC ANA in the long term, 10 male SD rats were treated with a single dose of either 7.0 mg/kg (*n* = 5) or 35.0 mg/kg (*n* = 5). The rats were purchased from SPF Biotechnology Co. Ltd. (Beijing, China). Animals were acclimated to the test facility for at least three days, during which their general health was checked by veterinary staff or other authorized personnel. Animals were kept in polycarbonate cages containing absorbent corncob bedding, with a maintained temperature range of +20–25 °C and a 12-h light/dark cycle. Animals had access to food and drinking water *ad libitum* during acclimation and the experiment. At the end of the study, the rats were euthanized by exposure to an increasing concentration of CO_2_. No abnormal clinical symptoms were observed during any of the experiments in the SD rats.

Anti-tumor efficacies of PO- and SC-administered ANA were observed *in vivo* using a patient-derived xenograft (PDX) of UZLX-GIST2B. The mice were purchased from Janvier Laboratories (Le Genest-Saint-Isle, France) and the UZLX-GIST2B model, carrying a *KIT* exon 9 mutation (*KIT*: p.A502_Y503dup), was developed in and used by the Laboratory of Experimental Oncology, KU Leuven, Belgium (Gebreyohannes et al., 2019). The selected model has previously been discovered to be sensitive to ANA administered PO (Pulkka et al., 2019). After a 1–2-week acclimation period, 7–8-week-old female NMRI *nu/nu* mice (weight range: 29–37 g) were inoculated bilaterally in both flanks under isoflurane anesthesia. The bilateral tumors were counted as individual tumors/events. The 34 mice were assigned to one of four treatment groups: (A) vehicle control (*n* = 8); (B) 2.5 mg/kg PO ANA (*n* = 8); (C) 35 mg/kg SC ANA (*n* = 9); or (D) 70 mg/kg SC ANA (*n* = 9). During acclimation and the experiment, mice were kept in polycarbonate cages containing bedding material (aspen shavings), a nest box, and cotton nesting material, at a temperature of +22 °C and a 12-h light/dark cycle. They had access to food and water *ad libitum.*

### Drug formulations

ANA hydrochloride monohydrate (CAS# 823178-43-4, batch no. NJ200423V, particle size 5 µm, purity ≥99% HPLC) was manufactured by CPL Sachse (Berlin, Germany), and the drug formulations were prepared in Avivia BV (Nijmegen, The Netherlands).

The SC formulation was prepared by dissolving micronized ANA (5 µm particle size) in an oily triglyceride ester of saturated caprylic and capric fatty acids and glycerol (Miglyol^®^ 810 N, IOI Oleo GmbH, Hamburg, Germany). The SC formulation concentrations were 0.2 mg/mL in the study comparing three administration routes in rats, 59.1 mg/mL in the long-term evaluation of SC ANA in rats, and 21 mg/mL in the efficacy study with mice.

In the IV formulation, the final solution contained 0.5 mg/mL ANA in 10% (v/v) DMSO, 5% (v/v) Cremophor, 2% (v/v) Tween 80, and 83% (v/v) acetate buffer (0.015 M), with a final pH of 8.05.

The PO formulation was obtained by first dissolving ANA in 10% ethanol and then vortexing and sonication, to a final concentration of 0.50 mg/mL.

### High-performance liquid chromatograph (HPLC) and tandem mass spectrometry (MS/MS) in analyzing SD rat plasma

To evaluate plasma concentration–time profiles, blood samples were collected from the jugular vein in EDTA tubes and stored at −70 °C until further analysis. To compare the three administration routes, blood samples were collected at 5 min, 15 min, 30 min, 1 h, 2 h, 4 h, 6 h, 8 h, and 24 h post-dose from rats that received IV and PO ANA. For those that received SC ANA, samples were collected at 0 min, 30 min, 1 h, 2 h, 4 h, 6 h, 12 h, and 24 h, followed by collection every three days until day 34 after dosing. In the second PK analysis in rats with a longer follow-up time, blood samples were collected at 0, 30 min, 1 h, 2 h, 4 h, 6 h, 12 h, 24 h, 3 d, 6 d, 9 d, 12 d, 15 d, 18 d, 21 d, 24 d, 28 d, 35 d, 42 d, 49 d, and 56 d after dosage.

SD rat plasma was analyzed for ANA, 3-OH-ANA, and RL603 concentrations by Pharmaron (Beijing, China). ANA, 3-hydroxy-anagrelide (CAS No. 733043-41-9, Cat. No. sc-206647, Santa Cruz Biotechnology, Inc., Dallas, TX), and RL603 (CAS No. 327602-34-6, Cat. No. sc-391661, Santa Cruz Biotechnology, Inc., Dallas, TX) were used as reference items in calibration standards and quality control (QC) samples.

The HPLC and the MS/MS (LCMS-8060) instruments were both from SHIMADZU (Kyoto, Japan), and the column used was a Raptor Biphenyl column (2.7 µm, 50 × 2.1 mm, Restek, Bellefonte, PA). The desired serial concentrations of working solutions were achieved by diluting analyte stock solution with 50% acetonitrile in water. Five microliters of working solutions (2, 5, 10, 20, 50, 100, 500, 1000, 5000, and 10,000 ng/mL) were added to 50 µL of blank plasma sample to achieve calibration standards of 0.2–1000 ng/mL (0.2, 0.5, 1, 2, 5, 10, 50, 100, 500, and 1000 ng/mL). QC samples at 0.5, 1, 2, 50, and 800 ng/mL were prepared independently of those used for the calibration curves. These QC samples were prepared on the day of analysis in the same way as calibration standards. Carbamazepine (CAS No. 298-46-4, Sigma-Aldrich, St. Louis, MO) was used as an internal standard for all analytes.

Altogether, 55 µL of standards, 55 µL of QC samples, and 55 µL of test samples (comprising 50 µL of plasma and 5 µL of blank solution) were added to 200 µL of acetonitrile containing an internal standard mixture to precipitate protein. The samples were then vortexed for 30 seconds. After centrifugation at 4000 × *g* (+4 °C) for 5 min, the supernatant was diluted threefold with water, and 2 µL of the diluted supernatant was injected into the LC/MS/MS system for quantitative analysis. The transitions were 256.00–198.90 *m/z* for anagrelide hydrochloride, 272.00–198.95 *m/z* for 3‐hydroxy anagrelide, 216.00–198.95 *m/z* for RL603, and 237.20–194.10 *m/z* for carbamazepine. Total run time per sample was approximately 2.5 min at a flow rate of 0.6 mL/min, using a gradient (0–2.00 min, 10–80% B) followed by isocratic elution (2.00–2.20 min, 80% B) and equilibration (2.21–2.50 min, 10% B).

Plasma drug concentration–time profiles were drawn by the average plasma concentrations at specific time points using Microsoft Excel (Redmond, WA). PK parameters, AUC_last_ (area under the plasma concentration–time curve from *t* = 0 h to the last measurable concentration), AUC_last_ (area under the plasma concentration–time curve from *t* = 0 h to infinity), *C*_max_ (peak plasma concentration), Cl (clearance), Cl/*F* (apparent clearance), *F* (bioavailability), MRT_inf_ (mean residence time extrapolated to infinity), *T*_max_ (time of *C*_max_), *T*_1/2_ (half-life), Vss (volume of distribution at steady state), and Vz/*F* (apparent volume of distribution) were calculated for each rat with the Phoenix™ WinNonlin^®^ Version 8.3 (WinNonLin; Certara, Princeton, NJ) software. Averages of each variable were used when PK values were compared.

**Table 2.** PK values calculated from ANA and its metabolite concentrations in plasma after IV, PO, or SC administration.

	IV	PO	SC
*Anagrelide*			
AUC_last_ (h*ng/mL)	261.05 ± 19.82	51.39 ± 7.09	167.19 ± 57.08
AUC_inf_ (h*ng/mL)	261.84 ± 19.91	59.28 ± 9.49	186.80 ± 62.02
*C*_max_ (ng/mL)	496.67 ± 17.46	17.99 ± 2.59	12.37 ± 3.14
*T*_max_ (h)	0.083 ± 0.00	1.00 ± 0.00	0.80 ± 0.27
*T*_1/2_ (h)	1.09 ± 0.22	10.05 ± 4.93	27.27 ± 24.44
Cl (mL/min/kg)	31.95 ± 2.52	na	na
Vss (L/kg)	0.97 ± 0.10	na	na
Cl/*F* (mL/min/kg)	na	718.05 ± 125.39	100.84 ± 37.37
Vz/*F* (L/kg)	na	633.58 ± 337.80	218.04 ± 148.85
*F* (%)	100	4.19 ± 0.70	33.47 ± 9.79
MRT_inf_ (h)	0.51 ± 0.08	6.38 ± 1.33	48.10 ± 22.70
*3-Hydroxy-anagrelide*			
AUC_last_ (h*ng/mL)	34.45 ± 19.06	17.29 ± 5.11	43.76 ± 54.40
AUC_inf_ (h*ng/mL)	34.76 ± 18.96	20.75 ± 7.44	na
*C*_max_ (ng/mL)	30.97 ± 12.45	5.43 ± 1.20	1.08 ± 0.48
*T*_max_ (h)	0.25 ± 0.00	1.00 ± 0.00	0.90 ± 0.22
*T*_1/2_ (h)	0.50 ± 0.14	4.65 ± 3.95	na
*RL603*			
AUC_last_ (h*ng/mL)	23.66 ± 13.81	24.94 ± 4.29	na
AUC_inf_ (h*ng/mL)	16.34 ± 4.54	34.76 ± 9.94	na
*C*_max_ (ng/mL)	12.31 ± 2.85	3.48 ± 1.01	na
*T*_max_ (h)	1.00 ± 0.87	1.67 ± 0.58	na
*T*_1/2_ (h)	0.98 ± 0.24	13.10 ± 5.29	na

AUC_last_: area under curve from *t* = 0 to last observation; AUC_inf_: area under curve from *t* = 0 to infinity; *C*_max_: peak plasma concentration; *T*_max_: time of *C*_max_; *T*_1/2_: half-life; Cl: clearance; Vss: volume of distribution at steady state; Cl/*F*: apparent clearance; Vz/*F*: apparent volume of distribution; *F*: bioavailability; MRT_inf_: mean residence time extrapolated to infinity; na: not applicable.

Data are presented as mean ± standard deviation. RL603 values were not calculated for SC ANA due to insufficient data points at concentrations above the limit of quantification.

### Efficacy study in a PDX UZLX-GIST2B

The rat PK studies indicated a prolonged MRT_inf_ and *T*_1/2_ with SC administration. Therefore, we reduced the SC dosing interval from twice daily (BID) in PO administration to every four days (Q4D) and increased the dose to either 35 or 70 mg/kg, expecting to achieve maximum anti-tumor effects. Although the efficacy of SC-administered ANA was unknown, we anticipated the experiment would last approximately 20 days, based on prior results where a BID PO dose of 5 mg/kg produced significant tumor regression in 11 days in the same PDX model (Pulkka et al., 2019). If the experiment had been extended to this duration, the SC doses used would have still remained well below ANA levels found to be toxic, though not lethal, in mice (Fleming & Buyniski, 1983). The number of tumors and mice are shown in Table 1.

**Table 1.** PDX UZLX-GIST2B model characteristics and treatment setup.

Model	UZLX-GIST2B
*Model characteristics*	
Origin	Patient derived
*KIT* exon 9 mutation	p.A502_Y503dup
Experiment duration	11 days
Treatment	No. of tumors/mice
A. Vehicle control 50 µL, Q4D SC	8/15
B. Anagrelide 2.5 mg/kg, BID PO	8/15
C. Anagrelide 35 mg/kg, Q4D SC	9/17
D. Anagrelide 70 mg/kg, Q4D SC	9/18

Q4D: every fourth day; BID: twice daily.

Before and during the treatments, tumor volumes (mm^3^) were determined by three dimensional measurements (*x* × *y* × *z*) with a digital caliper. The treatments started after tumors reached an average volume between 150 and 300 mm^3^, followed by randomizing the mice to the four treatment groups with an even tumor volume distribution per group. PO-treated mice received 2.5 mg/kg of ANA BID. Mice in the control group were injected with 50 µL of miglyol vehicle, and mice receiving SC ANA were injected Q4D with 35 mg/kg or 70 mg/kg into the loose skin between the shoulder blades (days 1, 5, and 9). During the experiment, tumor volumes were measured on days 1, 4, 8, and 11. In addition, animal well-being was visually monitored, and body weights were measured every day. Absolute tumor volume changes between the first and last days were considered as one of the measurements of treatment efficacy. When most of the tumors in the treatment groups reached the volume of <25 mm^3^ on day 11, the experiment was ended and mice were sacrificed by a pentobarbital (Vetoquinol, Lure, France) overdose, followed by cervical dislocation. Tumors were collected, weighed, and snap-frozen in liquid nitrogen, and livers of the mice were evaluated by visual inspection.

### Tumor sample processing

The tumor samples were stored at −70 °C until further processed. After quick thawing, larger samples were cut into smaller pieces depending on their size. For *KIT* mutational analysis, four randomly chosen samples (one sample per treatment group) were sequenced as previously described (Salmikangas et al., 2022). Briefly, DNA was extracted with QIAamp DNA Mini Kit (Cat. No. 937236, QIAGEN, NRW, Hilden, Germany), followed by PCR performed with FastStart™ Taq DNA Polymerase dNTPack kit (Cat. No. 04738381001, Roche, Basel, Switzerland). Samples were screened by Sanger-sequencing at the Institute for Molecular Medicine Finland (FIMM, Helsinki, Finland) with ABI3730xl DNA Analyzer (Thermo Fisher Scientific, Waltham, MA). From eight samples (two of each treatment group), a small piece was taken and crushed in 1 mL of cold RIPA Lysis and Extraction Buffer (Cat. No. 89900, Thermo Fisher Scientific, Waltham, MA), followed by rotation in +4 °C for 1 h. The samples were then centrifuged at 14,000 × *g* at +4 °C for 8 min, and the suspension was then collected.

The rest of the samples were placed in 10% formalin overnight. Tissues were then dehydrated through a series of increasing ethanol concentrations (70%, 96%, and 100%), twice for each concentration for 1 h each time, and in xylene twice for 30 min, before finally incubating in paraffin overnight at +60 °C. Tissues were embedded in paraffin to produce blocks. Next, sections (3.5 µm in thickness) were generated on microscope slides for hematoxylin and eosin (H&E) staining and baked at +60 °C for 1 h. Sections were also generated on positively charged slides for immunohistochemical staining.

### Histological evaluation

Histological responses in tumor tissue were evaluated based on the extent of necrosis, fibrosis, and myxoid degeneration observed in H&E-stained whole tissue sections. The grade of response for each sample was categorized as followed: grade 1 (0–10%); grade 2 (>10% and ≤50%); grade 3 (>50% and ≤90%); and grade 4 (>90%) (Antonescu et al., 2005).

### Immunohistochemical staining

The formalin-fixed paraffin-embedded tumor sections were immunohistochemically stained to detect PDE3A, KIT, SLFN12, and HLA-A (major histocompatibility complex, class I, A). PDE3A and KIT staining was performed as previously described (Pulkka et al., 2019; Toivanen et al., 2023). Staining conditions are given in Table S2. HLA-A was investigated to examine whether the PDE3A- and KIT-negative cells in SC ANA treated tumor samples were of human or mouse origin.

Slides were counterstained with hematoxylin. Interstitial cells of Cajal within healthy colon muscle tissue were used as positive control for PDE3A and KIT staining, and syncytiotrophoblasts in placenta tissue for SLFN12 (Figure S1). Control tissues were not included in HLA-A staining, given its ubiquity across human tissues. Staining intensities were scored as 0 (no staining in tumor cells), 1 (weak), 2 (moderate), or 3 (strong), except for HLA-A for which cells were only evaluated as positive or negative.

### Western blot

Western blotting was performed as previously described (Toivanen et al., 2023) with the following antibodies: PDE3A (1:1000, polyclonal rabbit, Cat. No. HPA014492, Sigma-Aldrich, St. Louis, MO), KIT (1:10,000, polyclonal rabbit, Cat. No. A4502, Agilent, Santa Clara, CA), SLFN12 (1:500, monoclonal rabbit, Cat. No. ab234418, Abcam, Cambridge, UK), alpha-actin (1:150,000, polyclonal rabbit, Cat. No. A300-491A, Bethyl Laboratories, Montgomery, TX), and anti-rabbit (1:10,000, polyclonal goat, Cat. No. 111-035-003, Jackson ImmunoResearch, West Grove, PA). Blotted proteins on PVDF membranes (Mini Format, Cat. No. 1704156, Bio-Rad, Hercules, CA), were detected with SuperSignal™ West Pico PLUS chemiluminescent substrate (Cat. No. 34580, Thermo Fisher Scientific, Waltham, MA) on UltraCruz^®^ autoradiography films (Cat. No. Sc-201697, Santa Cruz Biotechnology, Inc., Dallas, TX) using an automated X-ray film processor OPTIMAX 2010 NDT (PROTEC GmbH & Co. KG, Oberstenfeld, Germany). Western blotting was repeated twice, yielding similar results.

### Statistical analysis

Statistical analyses were performed using IBM SPSS Statistics 28.0.0.0 (Chicago, IL). *p* Values below .05 were considered statistically significant. Statistical significance of body weights, tumor volumes, and tumor weights between the treatment groups was analyzed using the independent samples Kruskal–Wallis test and Dunn’s pairwise tests adjusted with Bonferroni’s correction. To analyze differences in histological grades among all treatment groups, we utilized the Pearson *χ*^2^ test. Prior to analysis, the samples were divided into two categories based on their grades: low (grades 1 and 2) and high (grades 3 or 4).

## Results

### PK comparison between three administration routes and formulations

**Figure 1.**
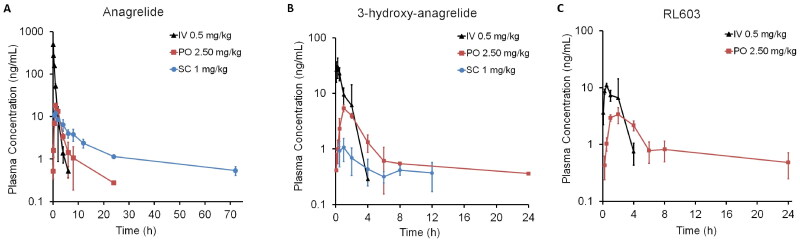
Plasma concentration–time profiles of (A) ANA and its metabolites, (B) 3-OH-ANA, and (C) RL603, after three different administration routes: IV, PO, or SC in rats. Metabolite concentrations quickly peaked after IV and PO administrations, but stayed relatively stable with SC administration. Data are presented as mean and standard deviation. RL603 was not detected above the quantifiable limit of 0.2 ng/mL after SC administration.

SD rats received a single dose of ANA either IV, PO, or SC, and plasma concentrations of ANA and its metabolites (3-OH ANA and RL603) were measured for 24 h after IV and PO, and up to 34 days after SC administration (Figure 1). As expected, IV dosing resulted in high *C*_max_ values (Table 2) detected immediately after administration, followed by rapid compound elimination within 6 h. In both PO and SC routes, ANA concentrations peaked at the 1-h time point (*T*_max_). The lowest ANA *C*_max_ value was observed with SC. However, while concentrations decreased approximately 90% by the 8-h time point after PO, the decline was more gradual with SC. Indeed, the ANA *T*_1/2_ nearly tripled with SC administration compared to PO and increased 25-fold compared to IV. ANA AUC_last_ was 36% lower with SC than with IV, while it increased threefold compared to PO. Furthermore, Cl/*F* and Vz/*F* were significantly lower for the SC route compared to PO, while MRT_inf_ was notably higher. Following SC administration, ANA concentrations were no longer detectable after the 72-hour time point, except for a few irregular observations near the quantification limit. Due to low ANA metabolite concentrations after SC dosing, a plasma concentration–time profile could be generated only for up to 12 h for 3-OH-ANA, and not at all for RL603. The ANA plasma concentration–time profile, along with the PK data, indicates that ANA delivered via the SC route exhibits controlled drug release compared to IV and PO, as well as improved bioavailability and a slower elimination rate compared to PO.

### SC ANA administration resulted in a long plasma exposure

**Figure 2.**
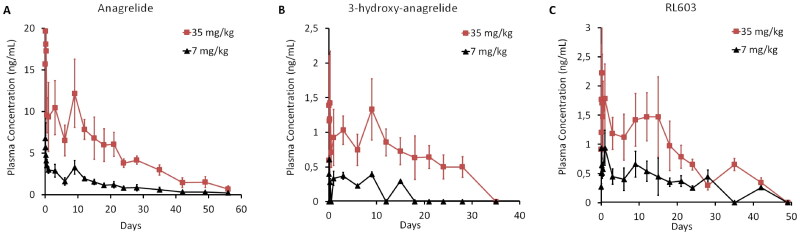
Plasma concentration–time profiles of (A) ANA and its metabolites, (B) 3-OH-ANA, and (C) RL603, after SC administration to SD rats of two different concentrations, 7 mg/kg and 35 mg/kg. Data are presented as mean and standard deviation.

Next, we investigated ANA and its metabolite plasma concentrations after a single SC dose (7 mg/kg or 35 mg/kg) for 56 days in SD rats. All plasma concentrations decreased in a sustained manner (Figure 2). The PK values (Table S1), AUC_last_, and *C*_max_, were in line with the dosage levels, and the *T*_max_ was typically observed at time points ranging from 1 to 4 h, varying greatly between individual rats. ANA *T*_1/2_ was similar across both doses, while the *T*_1/2_ values of ANA metabolites also exhibited considerable variation between individuals due to low plasma concentrations and irregularities.

### SC ANA efficiently suppressed tumor growth *in vivo*

During the *in vivo* experiment with UZLX-GIST2B, all treatments were well-tolerated, and only PO ANA resulted in significantly lower relative body weights, although still in the acceptable range (related samples Wilcoxon’s signed rank test, *p* = .016) (Figure S2). The twice-daily oral gavage also appeared to agitate the mice. The SC ANA treatment did not decrease body weights or result in other signs of toxicity affecting animal well-being. However, it did result in drug precipitates near the injection site (Figure S3), indicating slow release due to the incomplete release of ANA from the injection site. The experiment was terminated on day 11 due to efficient tumor volume regression, to enable tumor material collection for histological and molecular characterization. *KIT* mutation status (c.1504_1509dup, p.Ala502_Tyr503dup) was verified in all randomly chosen samples (one from each treatment group).

**Figure 3.**
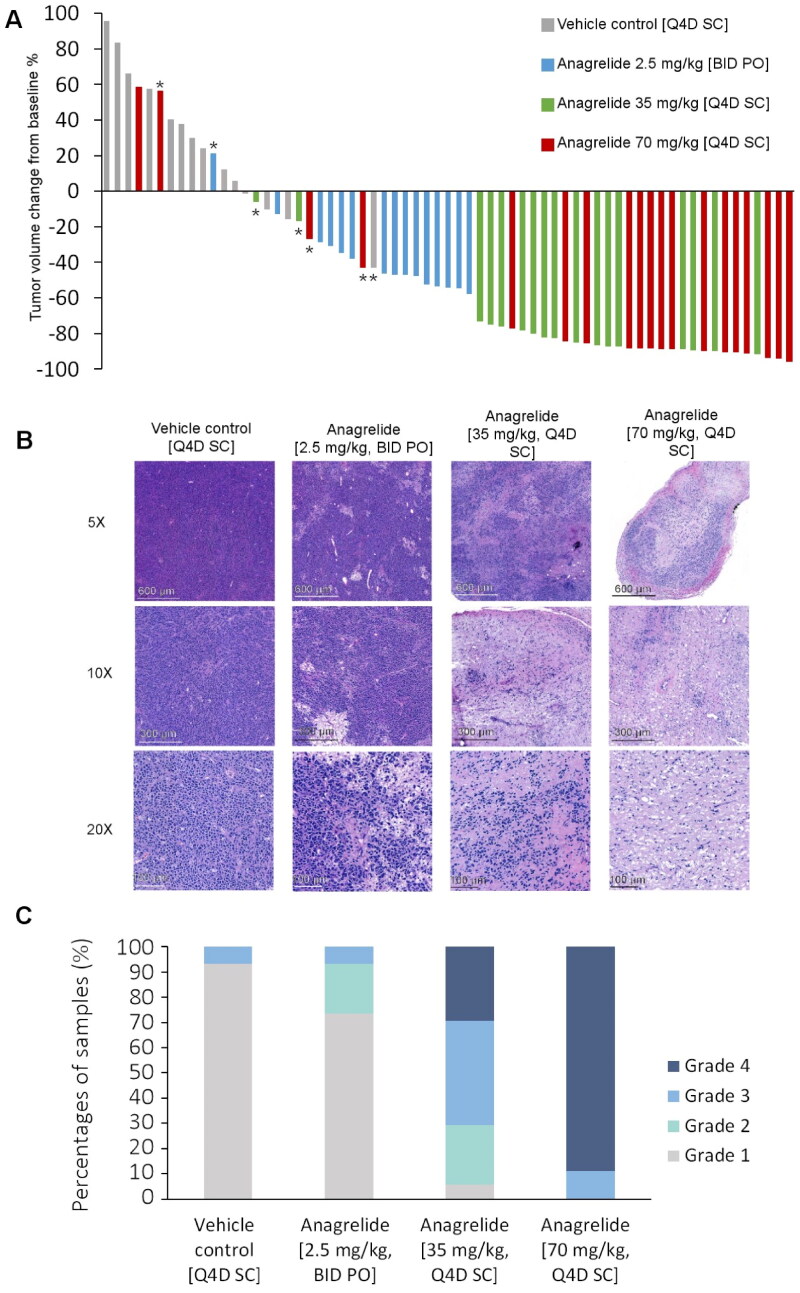
Treatment effects of ANA administered via PO or SC in a patient-derived GIST xenograft. (A) SC ANA treatment resulted in best tumor size reductions. *Tumors that were already very small at the beginning of the experiment. (B) Tumor samples were stained with H&E for histological response evaluation. (C) Treatment efficacy was graded based on the amount of necrosis, myxoid degeneration, and fibrosis in the H&E-stained tumor tissues.

Overall, 96% of all ANA-treated tumors shrank during the experiment, with the most pronounced tumor size reduction observed in SC ANA-treated tumors (Figure 3(A)). However, only the SC ANA-treated tumors had significantly smaller absolute tumor volumes compared to the control tumors on day 11 (Figure S4A) (the Kruskal–Wallis test, *p* < .001). The mean tumor volumes of the SC ANA-treated tumors were 25.18 ± 21.68 mm^3^ (35 mg/kg) and 20.43 ± 16.83 mm^3^ (70 mg/kg), versus 91.83 ± 66.41 mm^3^ of PO ANA-treated tumors and 194.30 ± 176.73 mm^3^ of the tumors in the vehicle control group. Tumors in the SC ANA-treated mice weighed significantly less than the tumors of the control and ANA orally treated groups (Figure S4B) (the Kruskal–Wallis test, *p* < .005). No significant differences were observed in tumor weights and volumes between the two SC ANA groups. Tumors that were small in the beginning of the experiment were observed in all treatment groups, and are indicated with an asterisk in Figure 3(A). These tumors were small (7.00–12.23 mm^3^) when the experiment started, indicating slow tumor growth rate or fibrosis of the tissue.

Both PO and SC ANA treatment mediated histological responses (Figure 3(B,C)). SC ANA treatment with the higher dose generated the strongest tissue responses (Pearson’s *χ*^2^ test, *p* < .001); 70% of the 35 mg/kg SC ANA samples were graded 3 or 4, whereas the equivalent value for the 70 mg/kg SC ANA treated tumors was 100%.

### SC ANA-treated tumors contained less PDE3A- and KIT-expressing tumor cells

**Figure 4.**
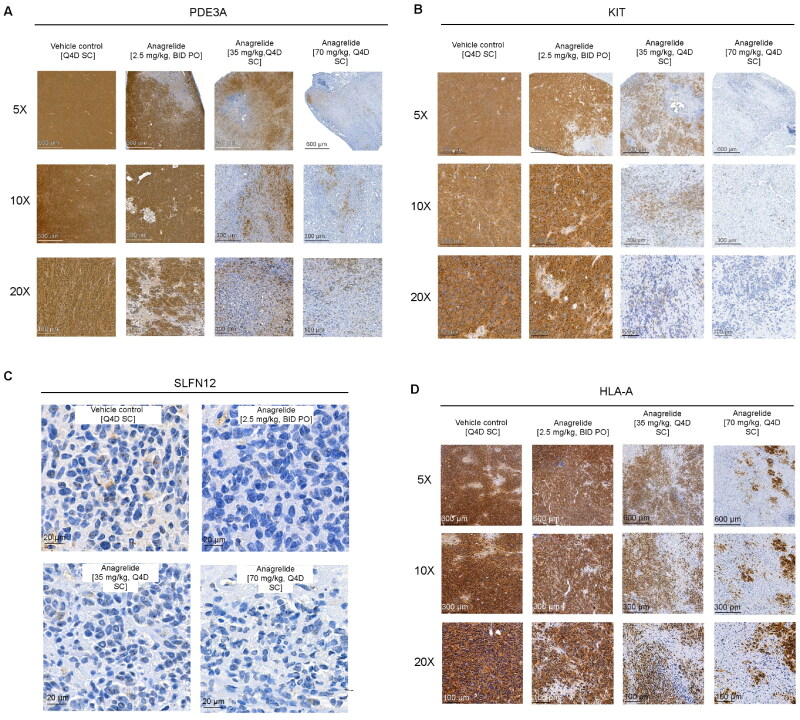
Protein expression was investigated in tumor tissue. (A) PDE3A was strongly detected with IHC from formalin-fixed paraffin-embedded FFPE tumor tissue samples, although the SC ANA-treated tumors exhibited less PDE3A-positive cells. (B) KIT was also strongly expressed in the tumor cells, and KIT-positive cells were observed less in SC ANA-treated tumor samples. (C) SLFN12 was weakly visible with IHC from all tumor samples. (D) SC ANA treated tumors contained multiple HLA-A negative cells, most probably originating from the host animal.

PDE3A and KIT were highly expressed in tumor cells in the control and PO ANA groups (Figure 4(A,B)). Noticeably fewer PDE3A- and KIT-expressing tumor cells were detected in SC ANA-treated tumors, indicating a reduction of tumor cells as a drug response in resected tissue samples. SLFN12 was faintly detected with immunohistochemistry (IHC) in the tumor tissue (Figure 4(C)). Protein expressions were also evaluated with Western blot from two randomly chosen samples of each treatment group (Figure S5). The results were consistent with the IHC findings, showing lower expression of PDE3A and KIT in the SC ANA samples, and overall low expression of SLFN12 in all samples.

HLA-A staining followed a similar pattern to that of PDE3A and KIT staining, suggesting that the tumor cells were efficiently eliminated with SC ANA treatment, allowing the host animal cells – in this case, mouse – to re-populate the remaining space.

## Discussion

ANA, an approved drug for treating essential thrombocythemia, shows promise against tumors that express elevated PDE3A levels, as in the case of GIST (Pulkka et al., 2019). However, fast metabolism of the drug following oral intake might require frequent dosing in clinical use to achieve optimal drug exposure in cancer cells and to promote tumor regression. The momentary high plasma concentrations and frequent administrations of therapeutic agents may also lead to unwanted side effects (Petrides et al., 2009). Overcoming these challenges is particularly difficult when dealing with compounds like ANA, which are characterized by short half-lives and water insolubility. Here, we investigated miglyol as an excipient for ANA via the SC route due to its dissolving ability. We compared PK of SC-administered ANA with those of PO and IV, assessed the long-term sustained-release in rats, and evaluated tumor regression efficacy of SC ANA in a patient-derived GIST xenograft mouse model.

The ANA plasma concentration–time profiles in SD rats, as well as the obtained PK values demonstrated that SC administration had the most sustained release pattern compared to PO and IV administration. These findings align with previous studies that observed increased AUC or *T*_1/2_ values, or both, when delivering compounds with poor solubility and short half-lives via the SC route compared to other administration routes (Lau et al., 1996; Marcucci et al., 2005; Moreau et al., 2011; Jeong et al., 2020; Wang et al., 2021). Although measuring plasma concentrations beyond the 24-h mark could have offered more precise PK values for the PO route, previous PO ANA PK studies in humans (Gaver et al., 1981; Petrides et al., 2009, 2018) suggest that metabolite concentrations would likely decline in a mono-exponential manner. Therefore, additional measurements are not expected to significantly increase estimates of systemic exposure to ANA following PO administration. Plasma concentration assessments over an extended period following SC dosage of either 7 mg/kg or 35 mg/kg revealed sustained ANA release throughout the entire 56-day period. Moreover, the PK values displayed a dose-dependent increase. Similar results have been reported where other therapeutic compounds were detected from plasma many days following a single SC dose in miglyol (Wicks et al., 1993; Wysham et al., 2016), and a dose-dependent increase in systemic exposure (Palin et al., 1982; Subhramanian et al., 2023) was documented. These points should be taken into consideration when proposing future preclinical and clinical studies.

As anticipated based on the enhanced bioavailability and prolonged clearance rate observed in rat PK studies, the SC ANA demonstrated good tolerability and effective anti-tumor activity in tumor-bearing mice, even with less frequent dosing intervals. SC ANA treatment significantly reduced tumor volumes in the UZLX-GIST2B mouse model, with high histological responses seen in 85% of the SC-treated tumors. Furthermore, HLA-A staining revealed that the remaining cells within the tumors were most likely host animal cells. These mouse cells presumably populated the space left after the tumor tissue degradation, contributing to the measured tumor volumes. The SC treatments were well tolerated. This is consistent with observations that SC drug delivery has often been associated with fewer adverse events due to its slower release profile (Moreau et al., 2011; Wang et al., 2021; Hadidi & Pazuki, 2022). Based on previous experiments (Pulkka et al., 2019), PO ANA has been known to result in tumor regression in UZLX-GIST2B. Hence, it was used as a control treatment in this experiment. The current PO dosage was half of that previously used, and the exhibited responses here were slightly weaker, as expected. Mice receiving PO ANA BID by gavage were more agitated compared to those receiving SC ANA, possibly explaining the slight decrease in body weights.

The main objective was not to directly compare the two administration routes, but to assess whether SC doses could achieve positive efficacy and tolerability with a reduced dosing frequency. If future studies aim to perform such comparisons, using equivalent dosages and the same vehicle for both routes are essential. Indeed, the dosages administered to SC-treated mice were significantly higher. Assuming the simple body surface area scaling factor of 12.3 (U.S. Food and Drug Administration, 2005), the dose of 2.5 mg/kg in mice corresponds to a human dose of approximately 14 mg/70 kg, which is 5–6 times the maximum recommended human oral dose. However, ANA PK in animals do not follow body surface area-based allometry. Additionally, since smaller animals typically have faster metabolisms (Rhomberg & Lewandowski, 2004) and mice often exhibit shorter elimination half-lives (Beconi et al., 2011; Gao et al., 2016; Waidyanatha et al., 2020; Mizuo & Mano, 2023), further preclinical studies investigating various SC ANA dosages and dosing schedules, supported by PK data, should be conducted to better estimate human-equivalent doses.

Sex-based PK differences also warrant further investigation, although ANA efficacy has been reported as sex-independent (Silver, 2005). Given that mice do not have a direct equivalent to human SLFN12 (Tilmisani et al., 2024), we believe that ANA does not demonstrate PDE3A modulator function in mice aside from its cytotoxicity on human-derived tumors. However, as sex can affect drug metabolism through sex-specific differences in cytochrome P450 enzyme expression (Kato & Kamataki, 1982), it is still possible that ANA efficacy in rodents may be indirectly influenced by metabolic differences. Additional PK and efficacy studies in female rats and male mice, respectively, could help clarify potential sex-specific differences in ANA metabolism.

In conclusion, SC-administered ANA delivered in a liquid lipid vehicle, miglyol, demonstrated a sustained-release PK profile and effective, dose-dependent GIST tumor cell elimination. These findings suggest a promising administration option for achieving prolonged systemic exposure of ANA. Future preclinical studies with SC ANA are warranted to evaluate metabolic differences between genders and facilitate the estimation of a starting dose in humans. These should be followed by clinical studies to assess whether SC ANA follows a sustained-release pattern while demonstrating efficacy against tumors, or in the treatment of essential thrombocythemia in humans.

## Supplementary Material

Supplementary material_Toivanen2024_Reviewed.docx

Supplementary Table S2_Toivanen2024.xlsx

Supplementary Table S1_Toivanen2024.xlsx

## Data Availability

The data will be shared upon reasonable request.
