# Supplementary material for: Pharmacokinetic profile and in vivo anticancer efficacy of anagrelide administered subcutaneously in rodents
Source: Drug Deliv. 2025 Feb 10;32(1):2463433. doi: 10.1080/10717544.2025.2463433 (PMC11816618; doi:10.1080/10717544.2025.2463433)
Supplement: Supplementary material_Toivanen2024_Reviewed.docx [file IDRD_A_2463433_SM8469.docx]

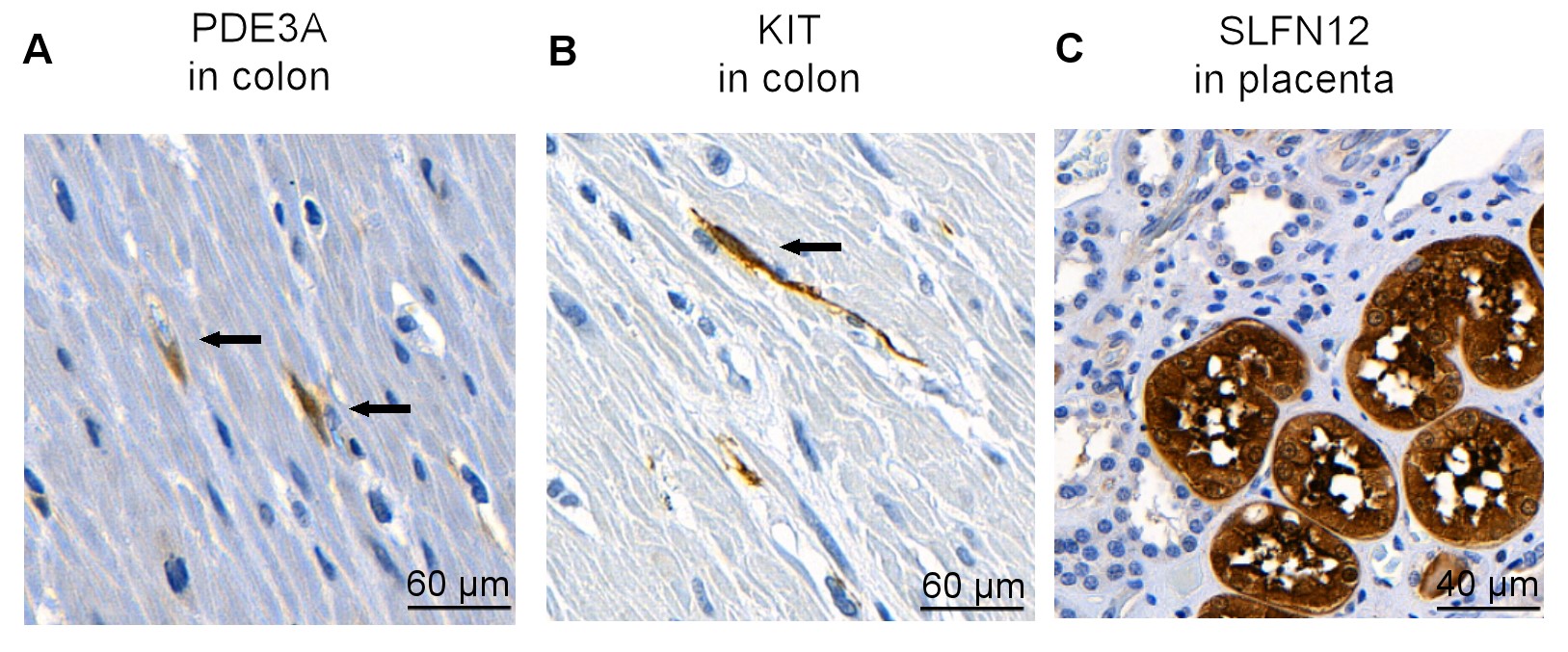


**Figure S1.** PDE3A, KIT, and SLFN12 immunohistochemical (IHC) staining of control tissues. Intestinal cells of Cajal (shown with arrows) in healthy colon tissue were used as a positive control for (a) PDE3A and (b) KIT IHC stainings. (c) Syncytiotrophoblasts in the placental tissue were used as a control for SLFN12 staining.


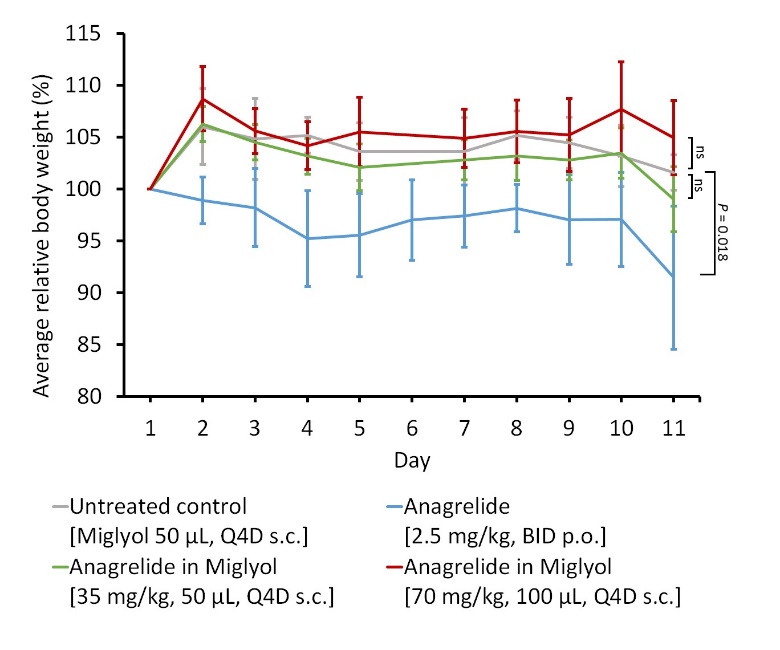


**Figure S2.** Relative body weights of mice during the efficacy experiment. Only PO treated mice showed a significant decrease in body weight. Data are presented as mean and standard deviation.


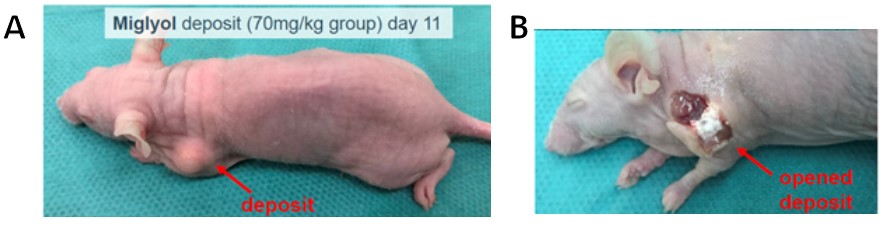


**Figure S3.** (a) Deposits were observed in the vicinity of the shoulder joint of SC ANA-treated mice. (b) When cut open, the deposits were noted to contain ANA precipitation.


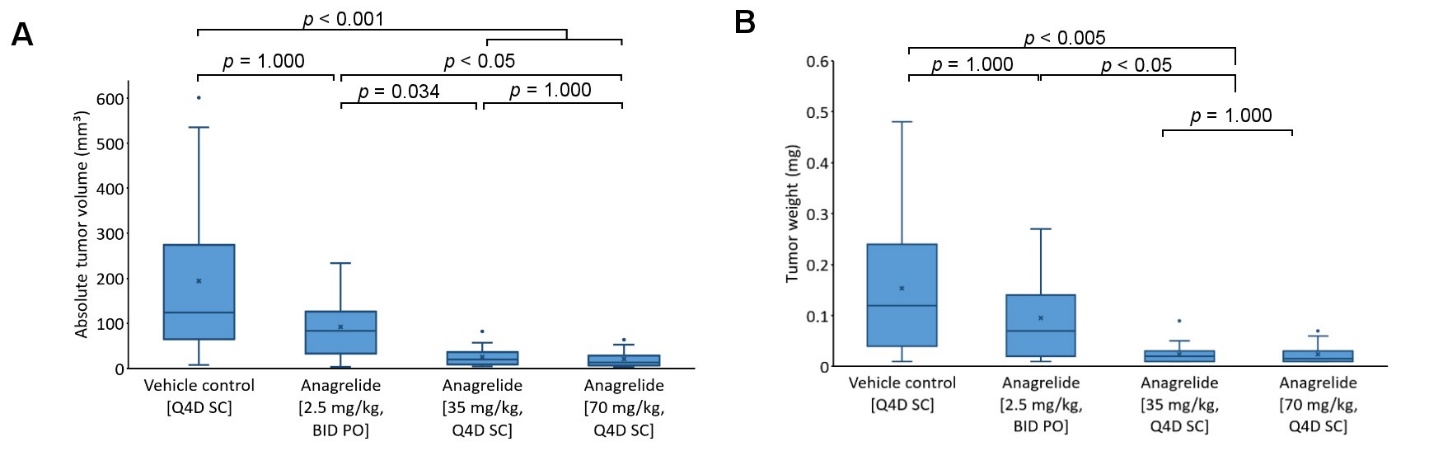


**Figure S4.** (a) Absolute tumor volumes and (b) tumor weights per treatment group. Data are presented as mean and standard deviation.


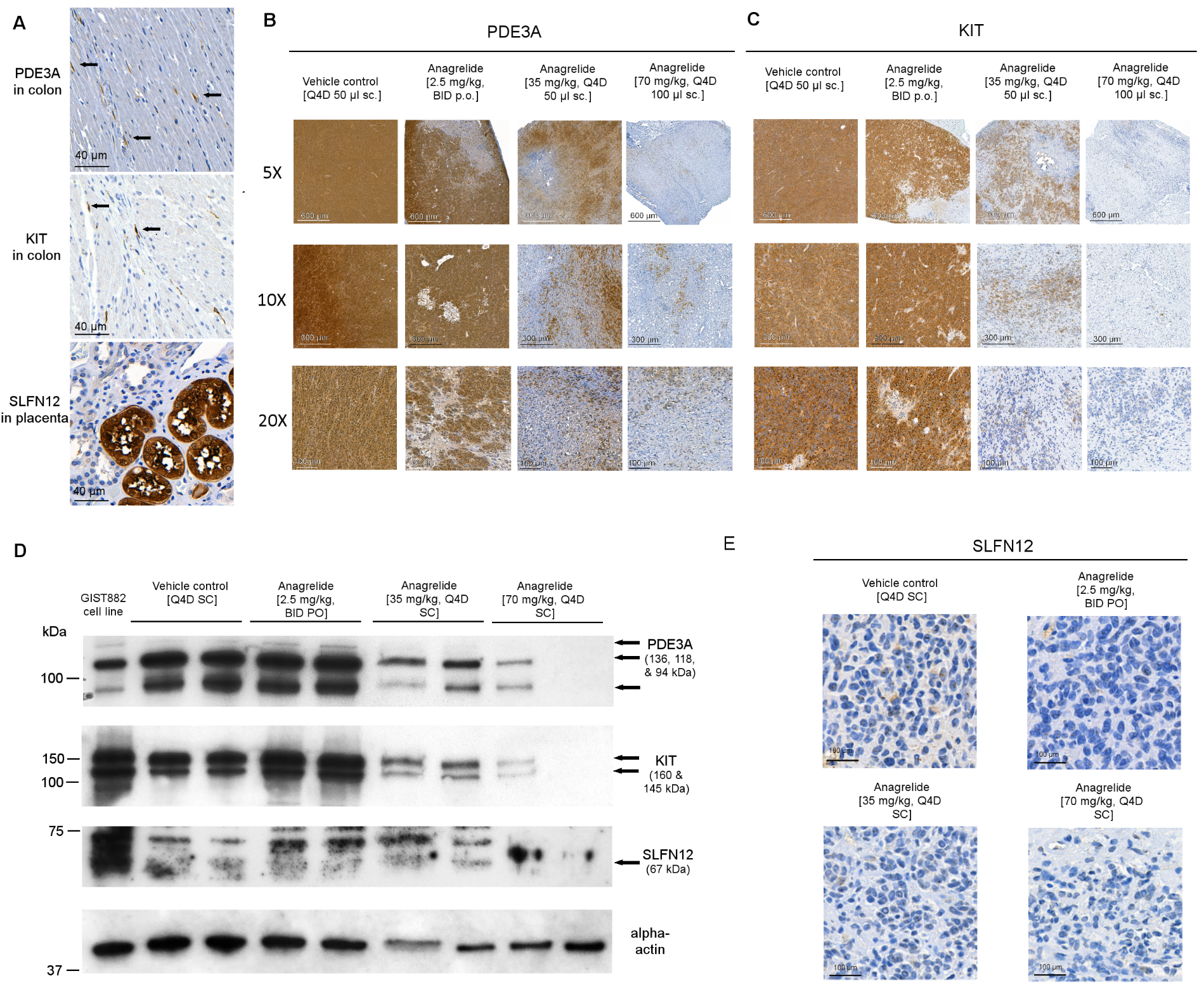


**Figure S5.** PDE3A, KIT, and SLFN12 Western blot results were in line with the IHC results. An ANA-sensitive cell line GIST882 lysate was used as a positive control.
